# Atorvastatin protects endothelium by decreasing asymmetric dimethylarginine in dyslipidemia rats

**DOI:** 10.1186/s12944-015-0041-2

**Published:** 2015-05-02

**Authors:** Dongdan Zheng, Qing Liang, FanFang Zeng, Zhuocheng Mai, Anping Cai, Ruofeng Qiu, Rulin Xu, Dongjuan Li, Weiyi Mai

**Affiliations:** Department of Cardiology, The First Affiliated Hospital of Sun Yat-sen University, 58 Zhongshan Road 2, Guangzhou, 510080 China; Department of Emergency Medicine, The First Affiliated Hospital of Guangzhou Medical University, Guangzhou, 510120 China; Department of Cardiology, Shenzhen Sun Yat-sen Cardiovascular Hospital, Shenzhen, 518000 China; Division of Biomedical Engineering, School of Engineering, Sun Yat-sen University, Guangzhou, 510060 China

**Keywords:** Dyslipidemia, Endothelium function, Statins

## Abstract

**Background:**

Asymmetric Dimethylarginine (ADMA) is an inhibitor of endogenous nitric oxide synthase, which is the key synthase for nitric oxide (NO) production. Whether statins could protect endothelium by reducing ADMA concentration is unclear, and whether this effect is associated with the dose of statins usage is also needed further studied.

**Methods:**

Dyslipidemia rat model was produced by giving high-fat and high-cholesterol diet for 8 weeks. Thereafter, low-dose (5 mg/kg body weight/day) and high-dose (20 mg/kg body weight/day) atorvastatin were orally prescribed for 4 weeks. Parameters of interest including lipid profiles, inflammatory and oxidative markers, NO production and plasma levels of ADMA and ADMA concentration of myocardium were evaluated. Liver enzymes and creatinine kinase (CK) were also detected for safety concern.

**Results:**

At baseline, all parameters were comparable between the sham and the dyslipidemia groups. At 8 weeks of dyslipidemia establishment, as compared to the sham group, body weight and lipid profiles were significantly elevated, and plasma levels of C-reactive protein (CRP), malondialdehyde (MDA) and ADMA were concomitantly increased in accompanying with NO reduction in the dyslipidemia groups. With 4 weeks of atorvastatin therapy, as compared to the control group, lipid disorders and NO production were improved, and plasma levels of CRP, MDA and ADMA were significantly decreased in the high-dose atorvastatin group. ADMA concentration of cardiac tissues was also significantly reduced in the high-dose atorvastatin group. Notably, there was a trend to similar effects which did not reach statistical significance in the low-dose atorvastatin group when compared to the control group. Liver enzyme and CK were comparable after 4 weeks of atorvastatin therapy between groups.

**Conclusion:**

In rats with dyslipidemia, atorvastatin therapy could reduce plasma level of ADMA and ADMA concentration in cardiac tissues, and these effects are associated with the dose of atorvastatin therapy.

## Introduction

Atherosclerotic cardiovascular disease (ASCVD) mainly including coronary artery disease and ischemic stroke is still the leading cause of morbidity and mortality worldwide despite great improvement has been achieved in the past decade [1]. As is well known that endothelial dysfunction is implicated in the development and progression of atherosclerosis and ASCVD [2,3], and previous many studies have shown that increased nitric oxide (NO) production by statins therapy contributed to better outcomes in experimental and clinical studies [4-6].

Asymmetric dimethylarginine (ADMA), a specific inhibitor of endogenous nitric oxide synthase (eNOS), is capable of decreasing NO production and may also accelerate atherosclerosis progression as suggested by previous studies [7-9]. For example, Sahinarslan A and colleagues reported that in subjects with stable angina, plasma ADMA concentration was positively correlated with coronary atherosclerotic score [10]. Moreover, increased plasma ADMA concentration might be a good indicator for predicting the severity of coronary artery disease [10]. Data from basic research revealed that in the setting of dyslipidemia, ADMA concentration was promoted which consequently leaded to endothelial dysfunction [11]. Outcome from subsequent clinical observational studies further supported the notion that ADMA might be a novel therapeutic target for preventing and treating atherosclerosis and ASCVD [7-9].

Knowingly, dyslipidemia is the major risk factor for atherosclerosis and ASCVD worldwide [12], and statins improved outcomes of ASCVD largely through modifying dyslipidemia as well as improving endothelial function [13,14]. However, in the early stage of dyslipidemia, whether statins could protect endothelium by reducing ADMA concentration is unclear yet, and whether this effect is associated with the dose of statins usage is also needed further investigation. Taken together, in order to address the above questions, dyslipidemia animal models were produced and then were treated with different doses of atorvastatin. We considered that the results from our present study could provide evidence regarding the potential therapeutic target of ADMA as a function of prevention and treatment of ASCVD in the future.

## Methods

### Animal model production

Sixty healthy male Sprague–Dawley rats weighing 200-220 g were obtained from the Experimental Animal Center of Sun Yat-sen University, Guangzhou, China. The study was approved by the Ethic Committee of Sun Yat-sen University. All animals received humane care in compliance with the Guide for the Care and Use of Laboratory Animals of the Institute of Laboratory Animal Resources, National Research Council.

After 1 week of accommodation, dyslipidemia animal model was produced in accordance to the previous described protocol [14]. Briefly, 15 rats were randomly selected and were used as the sham group, in which rats were given diet as usual, and the other 45 rats, evenly and randomly divided into 3 groups (n = 15), were used to produce dyslipidemia model as given with high-fat and high-cholesterol diet consisting of cholesterol (4.0%), cholic acid (0.4%), propylthiouracilum (0.3%) and lard (10.0%) for totally 8 weeks.

### Protocol of medical treatment

At 8 weeks of dyslipidemia model production, 5 rats from each group were randomly selected and sacrificed for evaluating the parameters of interest. The rest rats were given different medical treatments for totally 4 weeks. Briefly, rats with dyslipidemia were randomly assigned into the control group (given 3 ml of saline daily orally, n = 10), the low-dose atorvastatin group (5 mg/kg body weight/day of atorvastatin, prescribed orally, n = 10) and the high-dose atorvastatin group (20 mg/kg body weight/day of atorvastatin prescribed orally, n = 10). Rats in the sham group (n = 10) were orally given 3 ml of saline daily.

### Parameters of interest assessment

At baseline, 8 weeks after dyslipidemia model production, and 4 weeks after medical treatment, fasting venous blood in each group were drawn for parameters of interest assessment. Plasma levels of triglyceride (TG), total cholesterol (TC), low density lipoprotein-cholesterol (LDL-C), high density lipoprotein-cholesterol (HDL-C), and fasting blood glucose (FBG) were detected by Automatic Biochemistry Analyzer (Beckman coulter UniCel DxC 800 Synchron). Plasma levels of ADMA (enzyme-linking immune-absorbent assay kit, Wuhan Huamei Bioengineering Company, CSB-E13039r), malondialdehyde (MDA assay Kit, Nanjin Jianchen Bioengineering Institute, A003-2), C-reactive protein (CRP assay Kit, Nanjin Jianchen Bioengineering Institute, H151) and NO production (Total Nitric Oxide Kit, Beyotime, Haimen, China, S0023) were evaluated too. Briefly, the plasma level of ADMA was detected by enzyme-linking immune-absorbent assay in accordance to the manufacture’s instruction, and the detected wavelength is 450 nm. The intra-assay and inter-assay of coefficient variation were 9% and 11% respectively. At 8 weeks of dyslipidemia model production (5 rats from each group were randomly selected) and 4 weeks of medical treatment (the rest 10 rats in each group), after venous blood was drawn, the hearts of rats in each group were harvested for the evaluation of ADMA concentration in cardiac tissues (enzyme-linking immune-absorbent assay kit, Wuhan Huamei Bioengineering Company, CSB-E13039r). Cardiac tissues were homogenized and lysed with 500 μL of lysis buffer (50 mM Tris–HCl, pH 7.5; 5 mM EDTA; 250 mM NaCl; and 0.1% Triton X-100) containing 20 μL (10 mg/ml) of protease inhibitors PMSF. Concentration of protein was measured by Bicinchoninic acid (BCA) method. With respect to the safety of statins usage, liver enzymes (alanine aminotransferase, ALT and aspartate aminotransferase, AST) and creatinine kinase (CK) were also detected after 4 weeks of atorvastatin therapy.

### Statistical analyses

Continuous variable was presented as mean ± SD and compared by the Student’s *t*-test when data was normally distributed, otherwise compared by Wilcoxon rank-sum test. Categorical data was presented as percentage and compared by *χ*^2^ test. Statistical analyses were performed by using SPSS software version 18.0 (SPSS, Inc., Chicago, Illinois). A value of p <0.05 was considered significant.

## Results

### Baseline characteristics of rats in each group

In order to evaluate the changes of parameters of interest pre- and post-medical treatment, baseline characteristics of rats in each group were evaluated. As shown in Table 1, plasma levels of lipid profiles and FBG were comparable between groups at baseline. In addition, plasma levels of ADMA, MDA and CRP, and NO production were also detected and all the parameters were comparable between groups.

**Table 1:** **Baseline characteristics of rats**

**Variables**	**Sham (n = 15)**	**Control (n = 15)**	**Low-dose (n = 15)**	**High-dose (n = 15)**
Body weight (g)	215.51 ± 2.70	213.24 ± 3.62	216.45 ± 2.82	215.26 ± 3.33
TG (mmol/L)	1.03 ± 0.06	1.06 ± 0.08	1.03 ± 0.05	1.04 ± 0.07
TC (mmol/L)	3.45 ± 0.11	3.52 ± 0.14	3.36 ± 0.15	3.44 ± 0.13
LDL-C (mmol/L)	2.05 ± 0.10	2. 08 ± 0.12	2.06 ± 0.14	2.09 ± 0.12
HDL-C (mmol/L)	0.93 ± 0.04	0.91 ± 0.04	0.90 ± 0.05	0.93 ± 0.03
FBG (mmol/L)	4.06 ± 0.05	4.10 ± 0.08	4.08 ± 0.05	4.10 ± 0.08
NO (umol/L)	11.73 ± 1.05	12.02 ± 1.14	11.81 ± 1.09	10.98 ± 1.04
CRP (mg/L)	2.16 ± 0.32	2.28 ± 0.33	2.17 ± 0.22	2.32 ± 0.35
MDA (nmol/L)	0.87 ± 0.04	0.90 ± 0.06	0.89 ± 0.03	0.93 ± 0.05
ADMA (ng/mL)	42.52 ± 6.40	44.75 ± 5.33	43.90 ± 6.05	44.15 ± 5.20

### Changes of parameters of interest pre- and post-medical treatment

Parameters of interests before and after medical treatment were evaluated and compared between groups. As presented in Table 2, body weight and plasma levels of lipid profiles were all significantly increased in the dyslipidemia groups as compared to the sham group, suggesting that dyslipidemia model was successfully established. Of note, FBG level was also concomitantly increased, indicating that in the early stage of dyslipidemia, glucose homeostasis was already impaired. Moreover, parameters of inflammation, oxidation, and endothelial function were also profoundly deteriorated in the dyslipidemia groups. As shown in Table 2, plasma levels of CRP, MDA and ADMA were significantly increased while NO production was profoundly decreased in the dyslipidemia group when compared to the sham group (p < 0.05). Nonetheless, improvements were observed after 4 weeks of medical treatment. Notably, plasma levels of TG, TC and LDL-C were significantly reduced in the high-dose atorvastatin group as compared to the control group, and FBG level was also slightly reduced. Moreover, plasma levels of CRP, MDA and ADMA were significantly decreased, and NO production was profoundly increased in the high-dose atorvastatin group (p < 0.05). Briefly, improvements were also observed in the low-dose atorvastatin group. However, no statistical difference was observed when compared to the control group. After 4 weeks of atorvastatin therapy, no significant differences of ALT, AST and CK levels between groups were observed, suggesting that a short-term of high-dose atorvastatin was safe.

**Table 2:** **Changes of parameters of interest pre- and post-medical treatment**

**Variables**	**Sham**	**Control**	**Low-dose**	**High-dose**
**Pre-medical treatment (8 weeks of model production, n = 5 each group)**				
Body weight (g)	254.8 ± 6.6*	286.6 ± 7.0	290.3 ± 8.7	288.5 ± 10.4
TG (mmol/L)	1.15 ± 0.17*	1.79 ± 0.33	1.78 ± 0.32	1.76 ± 0.43
TC (mmol/L)	3.96 ± 0.26*	4.97 ± 0.34	4.96 ± 0.37	4.95 ± 0.45
LDL-C (mmol/L)	2.12 ± 0.24*	2.85 ± 0.31	2.91 ± 0.57	2.89 ± 0.44
HDL-C (mmol/L)	1.07 ± 0.12	1.11 ± 0.04	1.12 ± 0.04	1.10 ± 0.05
FBG (mmol/L)	4.12 ± 0.08*	4.49 ± 0.11	4.51 ± 0.12	4.50 ± 0.08
NO (umol/L)	12.67 ± 1.37*	9.65 ± 1.05	9.26 ± 1.08	9.44 ± 1.02
CRP (mg/L)	2.15 ± 0.42*	6.37 ± 0.55	6.52 ± 0.73	6.46 ± 0.52
MDA (nmol/L)	0.90 ± 0.06*	2.46 ± 0.18	2.57 ± 0.12	2.52 ± 0.19
ADMA (ng/mL)	43.37 ± 6.65*	60.68 ± 10.17	62.22 ± 8.17	62.30 ± 8.22
**Post-medical treatment (4 weeks of medical treatment, n = 10 each group)**				
Body weight (g)	277.3 ± 8.2*	301.2 ± 7.4	299.6 ± 10.3	300.6 ± 10.5
TG (mmol/L)	1.17 ± 0.22*	1.83 ± 0.42	1.67 ± 0.37	1.50 ± 0.29^#^
TC (mmol/L)	3.98 ± 0.33*	5.00 ± 0.62	4.81 ± 0.56	4.56 ± 0.36^#^
LDL-C (mmol/L)	2.13 ± 0.23*	2.87 ± 0.36	2.64 ± 0.52	2.43 ± 0.35^#^
HDL-C (mmol/L)	1.07 ± 0.14	1.10 ± 0.05	1.12 ± 0.03	1.10 ± 0.03
FBG (mmol/L)	4.14 ± 0.07*	4.56 ± 0.13	4.46 ± 0.15	4.41 ± 0.13
NO (umol/L)	12.88 ± 1.56*	9.03 ± 0.68	10.07 ± 0.64	10.85 ± 0.57^#^
CRP (mg/L)	2.13 ± 0.39*	6.43 ± 0.42	5.87 ± 0.16	5.35 ± 0.66^#^
MDA (nmol/L)	0.93 ± 0.07*	2.52 ± 0.15	2.29 ± 0.09	2.05 ± 0.07^#^
ADMA (ng/mL)	44.64 ± 7.08*	62.33 ± 10.08	58.75 ± 8.07	54.46 ± 9.15^#^
ALT (U/L)	21.43 ± 3.24	26.55 ± 5.21	24.47 ± 6.08	22.39 ± 3.76
AST (U/L)	24.36 ± 6.77	22.55 ± 4.09	21.56 ± 3.35	23.30 ± 6.12
CK (U/L)	18.24 ± 2.23	21.30 ± 2.08	20.07 ± 3.35	20.35 ± 2.56

Denote: *P < 0.05 versus other groups, ^#^P < 0.05 versus control group.

### Changes of ADMA concentration in cardiac tissues pre- and post-medical treatment

At 8 weeks of model production and at 4 weeks of medical treatment, after venous blood sample were drawn, cardiac tissues were harvested and used for ADMA concentration evaluation. As presented in Table 3, at 8 weeks of dyslipidemia model establishment, ADMA concentrations in cardiac tissues were promoted as compared to the sham group, nevertheless, after 4 weeks of atorvastatin treatment, when compared to the control group, ADMA concentration was significantly reduced in the high-dose atorvastatin group (p < 0.05) but marginally decreased in low-dose atorvastatin group (p = 0.078).

**Table 3:** **Changes of ADMA concentration in cardiac tissues**

**Variables**	**Sham**	**Control**	**Low-dose**	**High-dose**
**Pre-medical treatment (8 weeks of model production, n = 5 each group)**				
ADMA (μmol/mg protein)	35.15 ± 6.03*	48.53 ± 8.08	50.44 ± 10.52	49.67 ± 7.33
**Post-medical treatment (4 weeks of medical treatment, n = 10 each group)**				
ADMA (μmol/mg protein)	36.08 ± 7.43*	51.51 ± 8.26	47.26 ± 8.27	41.50 ± 7.08^#^

Denote: *P < 0.05 versus other groups, ^#^P < 0.05 versus control group.

## Discussion

Endothelial dysfunction induced by dyslipidemia is implicated in the development and progression of atherosclerosis. Statins, a potent lipid-modified agent, has potent effects on protecting endothelium through multiple mechanisms. Nonetheless, in the early stage of dyslipidemia, whether statins could reduce ADMA and thereby improve endothelial function has not been fully investigated yet. Moreover, whether this effect is associated with the dose of statins is also uncovered. Our present study showed that in rats changing from healthy to morbidity, high-dose of atorvastatin therapy conferred profound protection on endothelium as reflecting by increased NO production and reduced ADMA concentration in both plasma and cardiac tissues. Notably, endothelium-protection with low-dose of atorvastatin therapy did not achieve statistical significance when compared to the control group, suggesting that in the early stage of dyslipidemia, initiating high-dose of statins therapy might promptly prevent endothelial deterioration.

Currently, statins has been recommended as the cornerstone therapy for the primary and secondary prevention of ASCVD. Other than potent lipid-lowering effect, statins could exert other pleiotropic effects on cardiovascular systems [15,16]. For example, statins could ameliorate inflammation and oxidation in rats after acute myocardial infarction as reported by previous basic research [17]. Statins could enhance NO production by stabilizing the mRNA of eNOS [6,18]. Moreover, statins is capable of promoting endothelial progenitor cells migration and facilitating angiogenesis [19,20]. Consistent with previous reports [19,20], results from our present study revealed that atorvastatin therapy, with either low-dose or high-dose, could reduce plasma levels of CRP and MDA. NO production was also concomitantly enhanced with atorvastatin treatment. Nonetheless, with respect to the statistical significance, only high-dose of atorvastatin therapy achieved significant improvement on endothelial function as compared to the control group.

Knowingly, ADMA is a specific inhibitor of eNOS and many previous research have revealed that increased ADMA level was associated with adverse cardiovascular outcomes [21-23]. The underlying mechanism might be partially attributed to the adverse effects ADMA played on endothelium [24,25]. As is well known that through protecting endothelium, statins exerts its pleiotropic effects on cardiovascular system. Nevertheless, whether these benefits are associated with ADMA reduction is not fully investigated yet. Results from our present study revealed that atorvastatin therapy could reduce both the plasma level of ADMA as well as the ADMA concentration in cardiac tissues in rats with dyslipidemia after 4 weeks’ therapy. These benefits were enhanced with high-dose of atorvastatin regimen, while no statistical difference was observed between the low-dose of regimen and the control group, which we considered might be due to the short-term of atorvastatin therapy in the low-dose group as previous many studies have shown that the efficacy of statins therapy was associated with the duration of its usage [19,20]. The underlying mechanism associated with atorvastatin therapy on ADMA reduction was not investigated in our present study. However, to the best of our knowledge, we considered some mechanisms might be used to explain our findings. In the first place, it was possible that through modifying dyslipidemia, atorvastatin decreases plasma ADMA level. Data from our present study could provide evidence supporting this notion. As shown in Tables 1 and 2, when compared to baseline, plasma ADMA level was increased in accompanying with lipid disorders after 8 weeks’ high-fat and high-cholesterol diet treatment. In contrast, plasma ADMA level was concomitantly reduced when lipid disorders were improved by both low-dose and high-dose of statins therapy. Although there appeared to be a positive relationship between lipid disorders and ADMA change in our present research, we still could not definitely conclude that dyslipidemia was the unique and definitive cause of ADMA elevation. Secondly, since endothelium-protection by statins therapy is evident, it was therefore possible that atorvastatin could antagonize ADMA by directly up-regulating eNOS expression and promoting NO production. In the future, using eNOS knock-out animal model might be helpful to find out whether ADMA reduction by statins therapy was dependent on eNOS/NO signaling pathway. Thirdly, since statins has pleiotropic effects on different signaling pathway, therefore, it was possible that atorvastatin might regulate ADMA metabolism through promoting the activity of dimethylarginine dimethylaminohydrolase (DDAH) or regulating ADMA synthesis through inhibiting protein arginine N-methyltransferase (PRMT). In the future, it is warranted to investigate whether statins has effects on DDAH or PRMT. Last but not the least, the change of ADMA during the initiation and progression of atherosclerosis might be multi-factorial and atorvastatin might directly reduce ADMA level through modulating the gene expression or mRNA transcription.

## Conclusion

In summary, results from our present study show that in rats with dyslipidemia, atorvastatin therapy could reduce plasma level of ADMA and ADMA concentration in cardiac tissues, and these effects are more prominent by high-dose of atorvastatin therapy, suggesting that high-dose of statins therapy may confer rapid protection on endothelium, and future study is warranted to investigate whether this early benefit could ultimately turn into long-term clinical benefit.
